# The Future of Women and Heart Disease in a Pandemic Era: Let’s Learn from the Past

**DOI:** 10.3390/medicina57050467

**Published:** 2021-05-11

**Authors:** Suzanne Steinbaum

**Affiliations:** Mount Sinai Hospital, New York, NY 1468, USA; drsrsteinbaum@gmail.com

**Keywords:** pandemic and women, women and heart disease, psychological effects of pandemic, cardiac prevention, risk of heart disease in women

## Abstract

When the pandemic started in February, about 5 million women were running businesses. Just 2 months later, 25% of those businesses closed. Approximately 2.5 million women have lost their jobs or dropped out of the workforce since the pandemic, but that is just the start of the impact on women. Women have been disproportionately affected by the pandemic, as the brunt of homelife has fallen on them, and the psychosocial impact will inevitably have a physical impact. The pandemic has revealed the gender inequality that exists from the socioeconomic perspective, but soon we will see the impact from the medical perspective. Predictably, we know that the impact of stress and lack of self-care that women have had to endure heightens heart disease, already the number one killer of all women. Heart disease is 80% preventable based on the major risk factors: high cholesterol, high blood pressure, elevated sugar, obesity, smoking, sedentary lifestyle, and poor diet. But the psychological risk factors drive up biomarkers and the root causes of manifesting disease. Historically, women have been less diagnosed and treated, and less likely to receive lifesaving care in a timely fashion. The pandemic is sure to amplify these issues. Without mitigation and prevention, women’s hearts will suffer. We need to be aware of this now to prepare for the future potential of a significant increase in the incidence of women and heart disease.

When the pandemic started in February, about 5 million women were running businesses. Just 2 months later, 25% of those businesses closed. Approximately 2.5 million women have lost their jobs or dropped out of the workforce since the pandemic, but that is just the start of the impact on women [1]. Women have been disproportionately affected by the pandemic, as the brunt of homelife has fallen on them, and the psychosocial impact will inevitably have a physical impact. The pandemic has revealed the gender inequality that exists from the socioeconomic perspective, but soon we will see the impact from the medical perspective. As time goes on, we are going to see the impact of the stress and lack of self-care that women have had to endure, which will predictably lead to the number one killer of all women, heart disease. Heart disease is 80% preventable based on the major risk factors—high cholesterol, high blood pressure, elevated sugar, overweight, smoking, sedentary lifestyle, poor diet—but the psychological risk factors are often the reasons for driving the biomarkers up and the root cause of manifesting disease [2].

As we have identified the impact of the pandemic on the psychosocial risk factors, it becomes concerning to conceptualize what toll this will take on women’s hearts. The lack of equity that has been magnified in the lives of women socially may amplify the lack of equity that women have received when it comes to heart disease. We need to be concerned that the future of medicine for women will show a more significant impact on morbidity and mortality due to the disparities in care when it comes to women and heart disease. This is a critical time to address these issues, before we feel the impact.

## 1. The Historical Perspective

In 1964, the American Heart Association held the first conference for women on heart disease. It was titled “Hearts and Husbands” and the objective was to learn how to keep your husband’s hearts healthy. Research was done on men only at that point. Heart disease was a man’s disease. Caring for women’s hearts did not exist in the science, and it was not in the consciousness of the population that mattered the most: women themselves. 

In 1984, more women started dying of heart disease than men, and it all directed back to the lack of science. During the 20 years prior, women were not included in the major trials on heart disease from prevention to treatment, including diagnostic strategies, pharmacologic interventions, prevention and interventional procedures. This gap in research caught up to the reality, and the outcome was an increase in women’s mortality.

Then, in 1994, the Office of Women’s Health was created, which was the first governmental initiative pushing the agenda of research on women and heart disease. Yet, another decade passed before Go Red for Women through the American Heart Association began promoting research, education and awareness. When Go Red for Women began in 2004, only 30% of women were aware that heart disease was even a threat. These past two decades spurred more progress and more initiatives and research. Finally, things have started to change in demonstrable ways for women’s heart health, yet, still, only 38% of cardiovascular research participants are women [3].

The pervasive bias still exists. Women are less likely to be diagnosed, treated or to receive lifesaving care in a timely fashion. In fact, even when seeing their primary care doctors, cardiovascular disease is not the top concern for the women or their physicians. The primary care doctors placed a greater emphasis on weight issues and breast health over cardiovascular disease [4]. Yet, women are dying of heart disease more than all cancers combined [5].

## 2. The Medical Perspective

The medical community is falling short when it comes to women and their hearts. A recent global meta-analysis looked at women who had acute heart attacks to assess their outcomes. Not only did women have to wait longer for lifesaving procedures compared to male patients arriving to the hospital, but women also had increased mortality rates compared with males. In fact, there was up to a two-fold increased rate of mortality, repeat heart attack and stroke due to co-morbidities, due to delay in care and suboptimal treatment [6].

Even when looking at a younger population of women, previously considered to be immune from cardiovascular disease due to the protective effects of estrogen, there is an increase in cardiac events. The ARIC Community Surveillance Study found an increase in acute myocardial infarction hospitalizations in women less than 55 years old between 1995 and 2014. This is believed to be due to a decrease in the recognition of risk factors in this younger population, because of a lack of preventive strategies. These younger women had greater risk factors due to lifestyle choices, such as hypertension and diabetes, yet there was less likelihood for aggressive screening or treatment [7].

Other trials looked at this population of younger women and found a greater prevalence of diabetes, heart failure, chronic obstructive pulmonary disease, chronic kidney disease and obesity compared with the men.

As we are seeing what was previously considered a young healthy population, pregnant women, suffer increased rates of hypertension and diabetes, there is an increase in the risk of cardiovascular-disease-related deaths. In fact, women with obesity, hypertension and diabetes have the greatest risk of death during pregnancy [8]. The risk factors are the same risk factors that are driving the prevalence of heart disease in younger women higher and higher. 

## 3. The Equality of Care Perspective

Women physicians are growing in numbers, and although only 13 % of cardiologists are women, more than 50% of internal medicine residents are women. 

This is an important factor in women’s health. One systematic review evaluated eight studies examining patient outcomes based on the physician gender. One study showed that mortality rates for patients having a heart attack were highest for female patients treated by male physicians. On the other hand, if the physician was female then the mortality rates were the same between the male and the female patients. Male physicians who had more interactions and exposure to female patients, and even with female physicians, had more success in treating female patients [9].

To be better prepared, acknowledgment of these gender roles and then training doctors about them is critical. The bias is not permanent, once it is addressed. The time has come that we pivot from the old thinking. This is not the 1950s anymore, and women in the last year have suffered in a way that has not been seen before. 

## 4. The Psychological Perspective

My greatest concern comes down to the basics—who is suffering and who is dying from heart disease and how do we prevent it from happening in the future? 

We are coming off of a time filled with changes in lifestyle and huge psychosocial implications. Coping measures for emotional upheaval impacts behavior, resulting in overeating, sedentary behavior, poor diets, increased alcohol intake and overarching stress. The implications of this reaches into our lives, and these behavioral changes affect the essence of those risk factors that lead to heart disease. Obesity, high blood pressure and high cholesterol and elevated sugars are going to affect more and more women as we get through these days of the pandemic, and hopefully soon, to the other side. 

Depression itself is more common in women and doubles the risk of heart disease [10]. With stressful life events, chronic daily stressors and high levels of perceived stress, research has shown an increase in cardiovascular disease. In fact, work-related stress was associated with a 40% increased risk of cardiovascular disease, and perceived loneliness and social isolation increased the incidence by 50%. The connection between the mind and heart is significant: the more profound, the greater the burden of the psychosocial issues, like stress or depression. [11].

Not only has the emotional toll led to depression and anxiety, but for some, post-traumatic stress disorder. PTSD, defined as a “mental disorder that occurs after exposure to a potentially traumatic life event and is characterized by extreme levels of distress,” is associated with a 61% increased risk of coronary artery disease, with associations with traditional risk factors. Along with these, anger and hostility, depression and pessimism all have a role in cardiovascular outcomes. With declining mental health, healthy behaviors are less likely to be prevalent, with a reduction in exercise consistency and poor dietary choices [10].

As women have been juggling the traditional roles with increased work stress and decreased income, there is the potential for an increase in heart disease. Perhaps this impact has just begun, and we will start seeing the true burden in years to come. As a cardiologist, I know the toll will roll out increasingly over time and we will continue to see an uptick in heart disease in this same population that, during this intensely stressful time, was more forced to carry the load than ever before. 

For women, this mind–body connection is even stronger. Depression and stress may quickly negatively impact physical health with increased weight gain and diminished exercise. The cycle, which has always been a factor in women’s heart health is now heightened directly because of the pandemic. 

## 5. The Future Perspective

The long-term impact of the pandemic’s attack on women will long be seen in women’s hearts. 

As we look at the research behind the pandemic, it becomes a concerning issue to conceptualize the toll that this pandemic will take on women’s hearts. With the awareness of the psychological and the economic toll, it is essential to discuss the physical toll—no one has spoken of what we are to expect regarding women and heart disease in the future. As a cardiologist working in prevention for two decades, I am sadly confident that the statistics will soon show the impact that this pandemic has had on women and their hearts. 

As the demographic of heart disease changes, it is essential to evaluate and understand this population, the reality behind the increase in risk factors, and the reasons behind the increased risk. This younger group of women is the group of women who are going to be most significantly impacted in terms of their heart health [11]. We are at a critical time in understanding the profound impact of these risks and are lagging behind in fully addressing them. 

Much like the past thirty years in general when we slowly evolved from a “man’s disease” to the realization that heart disease is the number one killer of women, we are again unprepared for what is to come. 

This simple lack of acknowledgement can possibly foreshadow the possibility that women will suffer greatly due to their heart disease. There continues to exist that unconscious, intrinsic, inexplicable bias that is still is pervasive, except when women patients get to see women doctors [12].

## 6. A Better Way Forward

We can do better, across the board. We need to focus on the needs of women and the true issues at hand. We need to understand the psychosocial impact and the true devastation that COVID-19 has on our population of women, and most importantly, we need to anticipate and address the future. 

We have had our wake-up call. We have seen the statistics, and after decades of watching more women die of heart disease than all cancers combined, it is time we pay attention. We need to shift our focus from looking at each patient the same and open our understanding to who women have been during this historical time. They have been carrying the load emotionally, which is taking a toll physically. 

The doctors need to be trained and ready to understand the symptoms of heart disease in women, be able to assess the risks, and expediently provide care that could possibly save her life [13].

As a community, we need to get ready for the women whose hearts have suffered and understand that if we do not address their mental well-being and the effects it has on the overall health, we will continue to misdiagnose, undertreat and enable women younger and younger to become ill. 

Women have just lived through the ultimate test for their hearts with chronic daily stressors and high levels of stress. It is time we get this issue straight and be prepared. Not only has the essence of women’s lives been put on the line, but also their hearts. I hope we are ready for it when the time comes.
